# Effects of the Sigma-1 Receptor Agonist 1-(3,4-Dimethoxyphenethyl)-4-(3-Phenylpropyl)-Piperazine Dihydro-Chloride on Inflammation after Stroke

**DOI:** 10.1371/journal.pone.0045118

**Published:** 2012-09-18

**Authors:** Karsten Ruscher, Ana R. Inácio, Kristian Valind, Arman Rowshan Ravan, Enida Kuric, Tadeusz Wieloch

**Affiliations:** Department of Clinical Sciences, Division of Neurosurgery, Laboratory for Experimental Brain Research, Wallenberg Neuroscience Center, Lund University, Lund, Sweden; Julius-Maximilians-Universität Würzburg, Germany

## Abstract

Activation of the sigma-1 receptor (Sig-1R) improves functional recovery in models of experimental stroke and is known to modulate microglia function. The present study was conducted to investigate if Sig-1R activation after experimental stroke affects mediators of the inflammatory response in the ischemic hemisphere. Male Wistar rats were subjected to transient occlusion of the middle cerebral artery (MCAO) and injected with the specific Sig-1R agonist 1-(3,4-dimethoxyphenethyl)-4-(3-phenylpropyl)piperazine dihydrochloride (SA4503) or saline for 5 days starting on day 2 after MCAO. Treatment did not affect the increased levels of the pro-inflammatory cytokines interleukin 1 beta (IL-1β), tumor necrosis factor alpha (TNF-α), interferon gamma (IFN-γ), interleukin 4 (IL-4), interleukin 5 (IL-5), and interleukin 13 (IL-13) in the infarct core and peri-infarct area after MCAO. In addition, treatment with SA4503 did not affect elevated levels of nitrite, TNF-α and IL-1β observed in primary cultures of microglia exposed to combined Hypoxia/Aglycemia, while the unspecific sigma receptor ligand 1,3-di-o-tolylguanidine (DTG) significantly decreased the production of nitrite and levels of TNF-α. Analysis of the ischemic hemisphere also revealed increased levels of ionized calcium binding adaptor molecule 1 (Iba1) levels in the infarct core of SA4503 treated animals. However, no difference in Iba1 immunoreactivity was detected in the infarct core. Also, levels of the proliferation marker proliferating cell nuclear antigen (PCNA) and OX-42 were not increased in the infarct core in rats treated with SA4503. Together, our results suggest that sigma-1 receptor activation affects Iba1 expression in microglia/macrophages of the ischemic hemisphere after experimental stroke but does not affect post-stroke inflammatory mediators.

## Introduction

Stroke induces an inflammatory response, which includes the activation and accumulation of resident microglia and peripheral leukocytes in the ischemic hemisphere [1]. Activation is regulated by specific molecules such as the NLRP1 inflammasome complex [2], and is also affected by the tissue microenvironment such as pH changes or production of reactive radicals such as reactive oxygen species from damaged cells [3].

A variety of cytokines, neurotrophic factors, neuropeptides, as well as neurotransmitters alter the activation state of microglia/macrophages [4] which can be monitored by the expression of ionized calcium binding adaptor molecule 1 [5]. Microglia/macrophages might be benefical and tissue protective [6]–[8]. Under pathological conditions, however, overactivation of microglia in the injured brain may contribute to further tissue damage and aggravation of secondary neuronal loss at the site of injury [9].

Upon brain ischemia, microglia/macrophages release inflammatory cytokines such as TNF-α and IL-1β in models of stroke [10]. An increased cytokine production may propagate the formation of reactive gliosis with consequences for the re-establishment and formation of neuronal circuits and be unfavorable for recovery of lost neurological function after stroke. Previous studies have shown that activation of the Sig-1R reduces microglia activity [11], and application of the unspecific sigma receptor ligand 1,3-di-o-tolylguanidine (DTG) suppressed the release of TNF-α, IL-10, and nitric oxide in lipopolysaccharide activated cells. In addition, enhanced neuronal survival observed after delayed DTG administration following experimental stroke was attributed to a reduced inflammatory response in the ischemic hemisphere [12]. A reduction of cytokine release was also observed after treatment with SR31747A [13] and SSR125329A [14], ligands for sigma receptors in models of rheumatoid arthritis and sepsis, respectively. Together, these studies suggest an involvement of sigma receptors in the inflammatory response after experimental stroke.

The aim of the present investigation was to evaluate if short-term treatment with the highly specific Sig-1R agonist SA4503 affects mediators of inflammation in the ischemic hemisphere during the first week after experimental stroke.

## Materials and Methods

### Ethics Statement

All animal experiments were carried out with the approval of the Malmö-Lund ethical committee and followed the ARRIVE guidelines [15].

### Transient Rat Middle Cerebral Artery Occlusion (tMCAO)

Transient MCAO was induced as described previously [16]. In brief, male Wistar rats (325–350 g, HsdBrlHan, Harlan Scandinavia, Denmark) were housed under diurnal light conditions and were fasted for 12 h before surgery. During surgery, physiological parameters were measured and controlled within physiological limits (Table 1). Rats were anesthetized (initial 4% fluothane in N_2_O/O_2_ (70∶30), during surgery 2% fluothane in N_2_O/O_2_, Astra Zeneca, Sweden) and the right common carotid artery (CCA) and external carotid artery were occluded permanently, the internal carotid artery (ICA) was exposed and ligated. A nylon filament (top diameter 0.3–0.4 mm) was introduced into the ICA via a small incision into the distal end of the CCA and pleaded up to occlude the origin of the MCA. During occlusion, regional cerebral blood flow was monitored by an optical fiber probe (Probe 318-I, Perimed, Sweden) connected to a laser Doppler flowmeter (Periflux System 5000, Perimed, Sweden). After 2 h, the filament was withdrawn. The same procedure was performed in sham-operated animals but no filament was introduced into the ICA. In total, 40 rats were operated, thereof 7 animals died within the first 48 h before treatment start and one rat was excluded due to weight loss.

**Table 1:** Physiological parameters of rats subjected to tMCAO at the time of recirculation.

parameter	vehicle	SA4503 (0.5 mg/kg s.c.)
mean arterial pressure (mmHg)	118.2±11.5	109.5±8.4
temperature (°C)	36.74±0.3	36.69±0.3
pCO_2_ (kPa)	39.98±3.5	40.61±5
pO_2_ (kPa)	105.9±12.6	101.83±9.6
pH	7.43±0.03	7.43±0.04
glucose (mmol/l)	6.44±1.0	6.56±1.1
body weight (g)	326.0±9.2	333.58±18.2

Data are presented as means ± standard deviation. No statistical differences were observed between the treatment groups.

### Randomization and Treatment Protocols

In order to obtain an experimental group with severe motor deficits, all animals were tested at 48 h after tMCAO for sensorimotor function on the rotating pole test and only rats with a severe deficit were randomly assigned into treatment groups [17]. At this time point, the infarct development has subsided. Rats were excluded from the study if a score higher than 2 was obtained in the test. Sham operated rats were excluded if the test score was lower than 3. Randomization was carried out blinded for the investigators who performed the surgeries and behavioral tests. Every other rat received SA4503.

**Figure 1:**
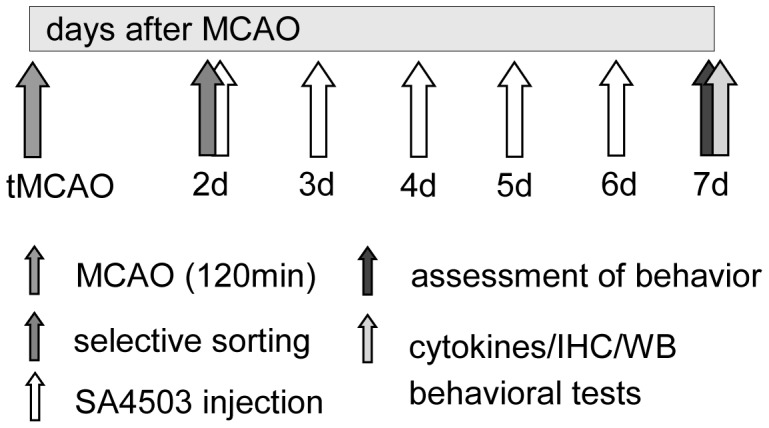
Experimental design.

Subsequently, starting on day 2 after MCAO, rats were injected daily with SA4503 (n = 12; 0.5 mg/kg bodyweight, s.c., AGY Therapeutics, South San Francisco, CA, USA) or vehicle (n = 10; saline) for 5 consecutive days (Figure 1), a dosage which has been demonstrated to improve lost neurological function after tMCAO [18]. In addition, 10 sham operated rats were injected (SA 0.5 mg/kg n = 5; vehicle n = 5) for the same time period.

### Infarct Size Measurement

Coronal brain sections (thickness 30 µm) were stained for the neuronal specific antigen NeuN (Millipore, Hampshire, UK dilution 1∶1000). The non-lesioned area of the infarcted hemisphere and the non-lesioned contralateral hemisphere were outlined and the infarct volume was calculated as described previously [17].

### Rotating Pole Test

Behavioral tests were always performed on awake rats between 6∶00 pm and 8∶00 pm and before injection. Sensorimotor function was evaluated using the rotating pole test as described previously. Briefly, rats traverse a rotating wooden pole (length 1500 mm, diameter 40 mm and elevation 700 mm) at 0, 3 and 10 rpm. Performance was video-recorded and evaluated by an experienced person blinded to the study using a 6 to 0 scoring system as described previously [17].

### Grip Strength Test

Forelimb strength was measured using the Grip Strength Test Meter GS3 (BIOSEB, France). Rats voluntary gripped a T-bar either with the healthy or paralyzed forelimb and the rat is pulled backwards. The maximum strength out of 3 trials was used in the data analysis as essentially described earlier [19].

### Microglia Cell Cultures and Combined Hypoxia/Aglycemia (H/A)

Microglia cell cultures were prepared from newborn rats (p2). After decapitation, meninges were removed from the tissue and cortices were mechanically dissected and digested in trypsin/EDTA solution (0.05% trypsin, 0.02% EDTA) at 37°C for 15 min. Digestion was stopped by addition of Dulbeccós minimal essential medium (DMEM) supplemented with 10% fetal calf serum (FCS), 1% penicillin/streptomycin, 2 mM L-glutamine and 0.1% glucose (culture medium) and thereafter the tissue was dissociated with a glass pasteur pipette (PBS). After centrifugation at 260×g for 2 min, cells were resuspended in fresh culture medium and seeded in 75 cm^2^ flasks. Cells were grown in culture medium and fed by complete medium change every third day. After 10 days, cultures were shaken for 1 h (250 rpm) in order to remove microglial cells. After pelleting, microglia were resown in subcultures and used for experiments 72 hours after plating. Combined H/A for 2 minutes was performed as decribed previously [20]. Duration of H/A has been evaluated in preliminary experiments in which microglia have been exposed to one, two and 5 minutes of H/A.

### *In vitro* Pharmacology

Immediately after H/A or normoxic control treatment, cells were treated with 10 µM SA4503 or 300 µM DTG. We prepared stock solutions of SA4503 in phosphate buffered saline (PBS, without Ca^2+^/Mg^2+^) and of DTG in ethanol and used 1∶100 dilutions in the experiments. Concentrations were chosen based on preliminary experiments showing toxic effects for SA4503 in concentrations above 10 µM, the DTG concentration was used as described previously [11]. For controls, only ethanol or PBS was added to the culture medium. Lipopolysaccharide (LPS) was applied in a final concentration of 1 µg/mL. For analyses we included at least N = 20 cultures from two independent preparations.

### Western Blotting

Proteins were extracted as described previously [16]. Ten micrograms of protein were separated on a 10% SDS polyacrylamide gel. Blocking was performed onto polyvinyldifluoride membranes using blocking buffer (20 mM Tris, 136 mM NaCl, pH 7,6, 0,1% Tween 20, 5% nonfat dry milk), and detected using a primary polyclonal antibody against Sig-1R (dilution 1∶1000, AGY Therapeutics, San Francisco, CA, USA), goat anti-Proliferating Cell Nuclear Antigen (PCNA; dilution 1∶2000, Santa Cruz Biotechnology, Santa Cruz, CA, U.S.A.), or mouse OX-42 (AbD Serotec, Kidlington, UK). After incubation overnight at 4°C, signals were obtained by binding of secondary HRP-linked antibodies (Sigma-Aldrich, diluted 1∶15000) recognizing the primary antibodies and visualized by exposing the membrane to a CCD camera (LAS1000, Fujifilm, Japan) using a chemiluminescence kit (Millipore, UK). Western blots for Iba1 were performed using the Snap i.d. system according to the manufacturer’s instructions (Millipore). The primary antibody was diluted 1∶1000 (Wako Chemicals, Neuss, Germany), the secondary antibody was diluted 1∶5000 (anti-rabbit HRP, Sigma-Aldrich) before exposing to the CCD camera. Membranes were stripped and reprobed for β-actin (Sigma-Aldrich, Germany, and diluted 1∶25000). After densitometric analysis, expression of Sig-1R and Iba1 was calculated as percentage of β-actin expression.

### Immunohistochemistry

Brain sections (thickness 30 µm) from 4% paraformaldehyde-perfused animals were gently washed three times in phosphate buffered saline (PBS, without Ca^2+^/Mg^2+^) and quenched (3% H_2_O_2_, 10% methanol) for 15 minutes. After blocking with 5% normal horse serum in PBS supplemented with 0.25% Triton X-100 for 60 minutes, the sections were incubated with a rabbit polyclonal Iba1 antibody (Wako Chemicals, Neuss, Germany, and diluted 1∶500) at 4°C over night followed by a secondary biotinylated horse anti-rabbit antibody (Vector Laboratories, CA, diluted 1∶200). Visualization was achieved via the Vectorstain ABC Elite kit using 3,3-diaminobenzidine/H_2_O_2_ (Vector). Omission of the primary antibody served as a negative control.

### Immunofluorescence

Brain sections from 4% paraformaldehyde-perfused animals were washed three times in phosphate buffered saline (PBS, without Ca^2+^/Mg^2+^). Thereafter, blocking was performed with 2% normal horse serum in PBS supplemented with 0.25% Triton X-100 for 60 minutes. For co-localization of proteins, the following antibodies were used: polyclonal rabbit anti-OX-42 (a general marker for microglia/macrophages, 1∶2000), polyclonal rabbit anti-Iba1 (1∶800, Wako Chemicals), and polyclonal rabbit anti sigma-1 receptor (1∶200, AGY Therapeutics, CA, USA). After overnight incubation at 4°C, cells were incubated with appropiate donkey secondary antibodies (Cy3 conjugated donkey anti-mouse antibody, biotinylated horse anti-goat antibody, both diluted 1∶200, Jackson Laboratories). Sections exposed to the secondary biotinylated horse anti-goat antibody before were further incubated with a Alexa 488 streptavidin conjugate (1∶200) at room temperature for 60 minutes. Fluorescent signals were visualized using a confocal microscopy system (LSM510 Zeiss, Germany).

### Iba1 Immunoreactivity

Immunofluorescence analysis for Iba1 was performed on 30 µm thick coronal brain sections from vehicle and SA4503 treated rats (level –1.8, in relation to bregma). Primary antibody was detected by a donkey anti-rabbit Cy3 conjugate (Jackson ImmunoResearch, Suffolk, UK). Micrographs were acquired in monochromatic 4-fold magnification covering the entire ischemic hemisphere within each section using a Nikon Eclipse 80i microscope and the NIS-Elements software (Nikon, Solna, Sweden) under standardized conditions essentially as described previously [20]. A final tile picture for each section was generated (NIS-Elements software, Nikon) and the detectable fluorescence signals in defined indicated areas of the ischemic hemisphere of each picture were encircled, and the average intensity level (0 to 255) per pixel was determined using the ImageJ software (National Institutes of Health), and denominated as average immunoreactivity. The areas were defined as following: 1. the apical cortical region of the infarct core adjacent to the peri-infarct area determined by NeuN^+^ cells in subsequent brain sections, 2. the cortical infarct core adjacent to the peri-infarct area adjacent to the corpus callosum, 3. the most apical part of the striatum adjacent to the corpus callosum, 4. the lateral part of the striatum bordering the corpus callosum.

### Multiplex Enzyme-linked Immunosorbent Assay and Griess Assay

Levels of the cytokines IL-1β, TNF-α, IFN-γ, IL-4, IL-5, and IL-13 were measured by ELISA according to manufacturers instructions using a SECTOR Imager 6000 reader (Mesoscale, Gaithersburg, MD, USA). Nitrite concentrations in the supernatant medium were measured by a commercial assay according to manufacturers instructions (Promega Corp., Madison, WI, USA).

### Statistics

The Western blot results and infarct volumes are presented as means ± SD and analyzed by t-test. Statistical analysis on cytokine data were performed by one-way ANOVA and posthoc Bonferroni correction. A p-value of p<0.05 was considered as significant.

## Results

### Effect of SA4503 Treatment on the Level of Pro-inflammtory Cytokines in the Ischemic Hemisphere

**Figure 2:**
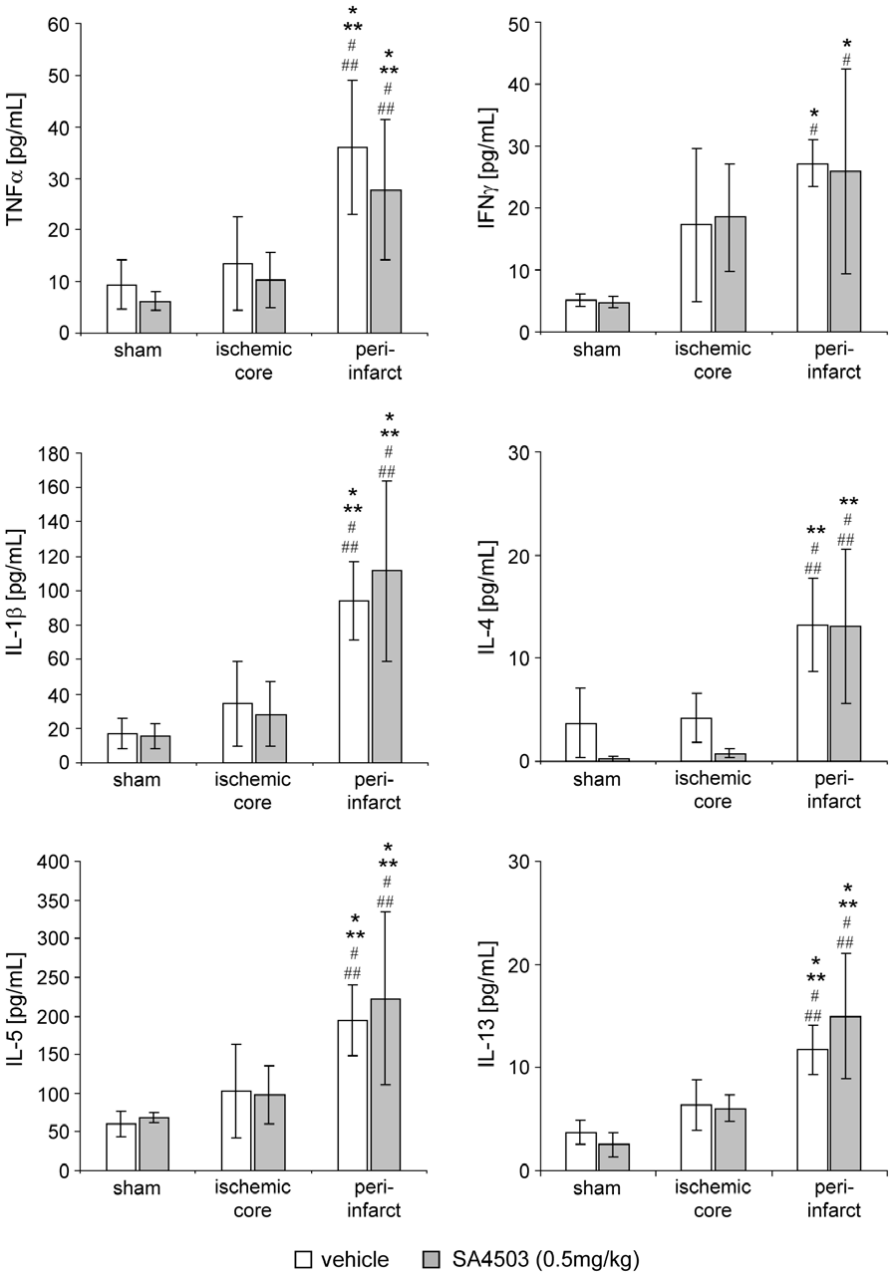
Analysis of cytokine in the ischemic hemipshere. Levels of intraparenchymal TNF-α, IFN-γ, IL-1β, IL-4, IL-5 and IL-13 of the ischemic core and peri-infarct region were measured from ischemic animals treated with saline (n = 8, white bars) or SA4503 (0.5 mg/kg, s.c., n = 8, gray bars) and of cortices from sham treated animals (saline n = 3; SA4503, 0.5 mg/kg s.c., n = 3) by multiplex ELISA using a SECTOR Imager 6000 reader. Data are presented as means ± sd. Statistical analysis was performed one-way ANOVA and posthoc Bonferroni correction (*p<0.05 vs sham saline, **p<0.05 vs ischemic core saline, #*p*<0.05 vs sham SA4503, ##*p*<0.05 vs ischemic core SA4503).

Levels of the pro-inflammatory cytokines IL-1β, TNF-α, and IFN-γ were significantly elevated in the ischemic hemisphere considered as hallmarks of inflammation after stroke (Figure 2) [21]. To evaluate if Sig-1R activation has an effect on these cytokines rats were injected with the specific agonist SA4503 for five days. As shown in Figure 2, SA4503 treatment did not change increased levels of cytokines. However, increased levels for IL-1β, TNF-α, and IFN-γ were detected in the peri-infarct area, the levels were unchanged in the infarct core. Here, cytokine levels were not significantly different to levels found in untreated (saline) or treated (SA4503) sham operated animals. We also observed increased levels for interleukin 4 (IL-4), interleukin 5 (IL-5), and interleukin 13 (IL-13) in the peri-infarct area after MCAO, hence unaffected by treatment with SA4503 (Figure 2).

**Figure 3:**
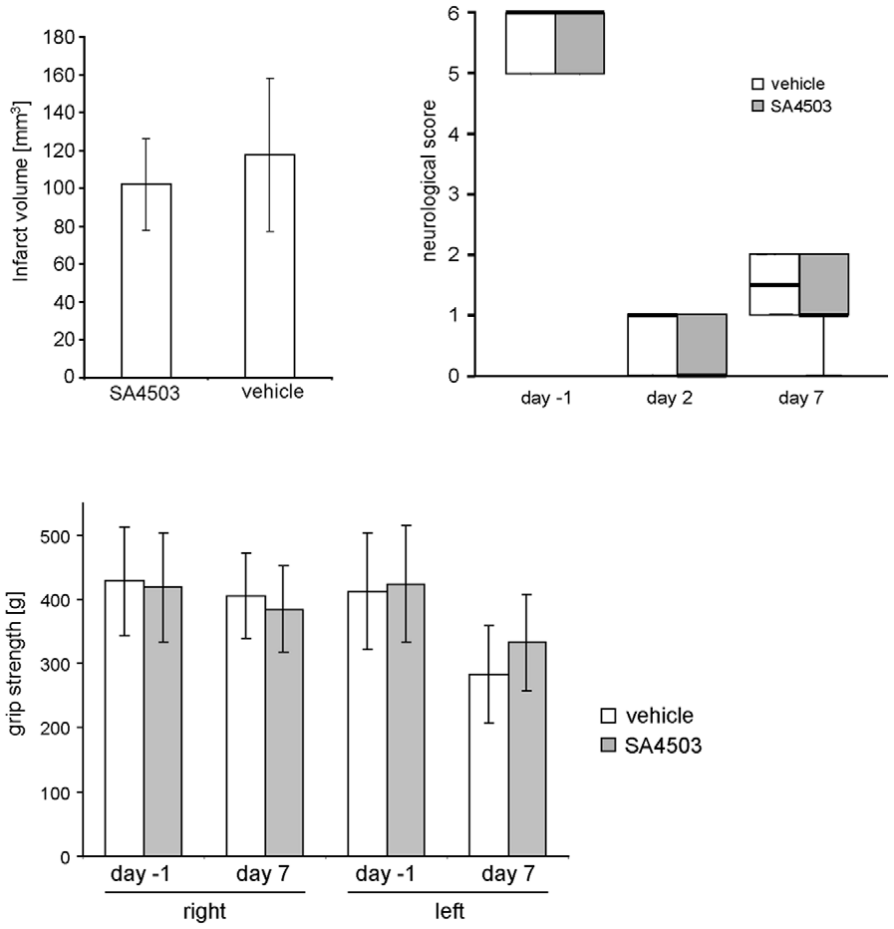
Effect of SA4503 on infarct volume and functional recovery after MCAO. (**A**) Infarct volume from vehicle (saline; n = 8) and SA4503 treated (0.5 mg/kg; n = 8) rats at 7 days following MCAO. Infarct volumes are calculated indirectly (nonlesioned contralateral hemisphere minus nonlesioned area of the ischemic hemisphere). No statistical difference was obtained between the treatment groups. (**B**) Evaluation of sensori-motor function in saline (vh, n = 10) and SA4503 treated (0.5 mg/kg, n = 12) rats at the indicated times before and after MCAO. Animals were tested on the rotating pole at 10 turns per minute to the left. Similar results were obtained with 10 turns to the right (data not shown). No statistical difference was obtained between the treatment groups at all time points. (**C**) Forelimb strength of the non-paralyzed and paralyzed forelimb was measured by a grip strength test meter one day before and at day 7 following MCAO (MCAO vehicle n = 10; MCAO SA4503 n = 12); vh – vehicle, SA – SA4503.

We have measured the infarct volume of rats treated either with saline (n = 8) or SA4503 (0,5 mg/kg; n = 8). Seven days after MCAO, animals in both treatment groups showed similar infarct volumes (vehicle: 117. 7±40.2 mm^3^, SA4503∶102.1±24.2 mm^3^) (Figure 3 A). The data corroborate a persistent inflammatory response in the ischemic hemisphere characterized by activation of immune cells and increased cytokine production that was not affected by Sig-1R activation. Importantly, Results were not confounded by different infarct volumes.

Also, no differences were found in recovery of lost neurological function between the treatment groups after MCAO. As shown in Figure 3 B, rats were unable to traverse the rotating pole before randomization into the treatment groups. At day 7 after MCAO, a partial recovery was observed, however, SA4503 treatment did not improve performance. Similar results were obtained by a standardized forelimb grip strength test. Here, the strength of the forelimb correlates with the grade of paralysis. Figure 3 C shows no differences of grip strength in the right non-paralyzed forelimb after MCAO. In contrast, grip strength was reduced in the left paralyzed forelimb compared with the strength of the forelimb obtained 1 day before MCAO (day –1∶411.9 g, day 7 saline: 282.8 g). Despite slightly better performance compared to saline treated animals after MCAO, rats subjected to MCAO did not benefit in this test from treatment with SA4503 (day 7 SA4503∶333.1 g) (Figure 3 C). Results corroborate that longer treatment periods are needed to obtain an improvement of lost neurological function after MCAO [18].

### SA-4503 does not Affect the Production of Nitrite and Pro-inflammatory Cytokines in Microglia after Combined Hypoxia/Aglycemia (H/A)

**Figure 4:**
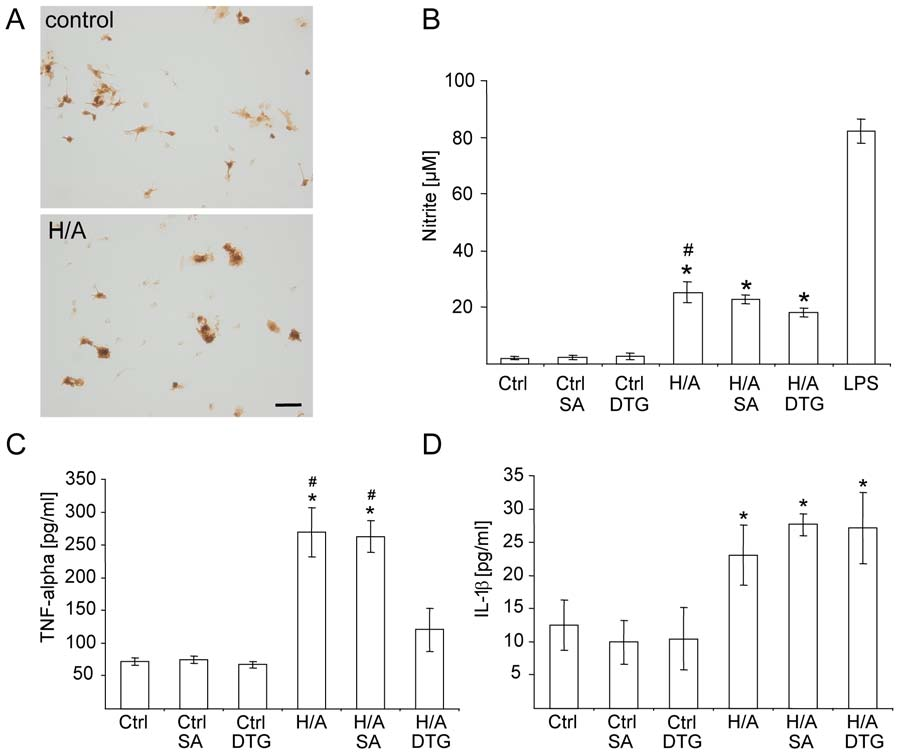
Effects of SA4503 and DTG on microglia after Hypoxia/Aglycemia. (**A**) Iba1 expressing microglia 24 h after normoxic control or combined H/A. Scale bar 10 µm. (**B**) Nitrite concentrations in the supernatant medium 24 h after stimulation as indicated in the figure. SA4503 (SA, 10 µM) and DTG (300 µM) were added to the culture medium immediately after H/A or normoxic control stimulation, respectively. Stimulation with lipopolysaccharide (LPS, 1 µg/mL) served as control. Abbreviations: ctrl – normoxic control, H/A – Hypoxia/Aglycemia. Data are presented as means ± SEM, *p<0.01 vs all control stimulations, #p<0.05 vs H/A DTG, one-way ANOVA and posthoc Bonferroni correction. **(C)** Levels of TNF-α and IL-1β after control or H/A stimulation and respective treatments (SA4503, 10 µM; DTG, 300 µM) for 24 hours. Data are presented as means ± SEM, n = 20 each experimental condition, *p<0.01 vs all control stimulations, #p<0.05 vs H/A DTG, one-way ANOVA and posthoc Bonferroni correction.

Immediately after a sublethal episode of H/A, microglia were treated either with SA4503 (10 µM) or DTG (300 µM). After 24 h, supernatant culture medium was analyzed for nitrite production and release of the pro-inflammatory cytokines IL-1β and TNF-α. As shown in Figure 4 A, Iba1 positive microglia changed into a more amoeboid morphology after H/A. Moreover, H/A induced a significant increase in nitrite production (control: 2.1±0.65 µM, H/A: 25.3±3.70 µM) which was not affected by tretament with SA4503 (22.9±1.71 µM), however, reduced after DTG treatment (18.2±1.49 µM) (Figure 4 B). In addition, DTG significantly reduced the release of TNF-α (120.19±32.96 pg/mL; H/A alone 269.21±37.87 pg/mL), whereas SA4503 had no effect on TNF-α levels in the supernatant medium after H/A (262.74±24.22 pg/mL). SA4503 and DTG treatment did not affect elevated levels of IL-1β after H/A (H/A 23.07±4.47 pg/mL, H/A SA4503 27.64±1.61 pg/mL, H/A DTG 27.10±5.32 pg/mL) and had no effect on the release of cytokines after normoxic control treatment (Figure 4 C). Results support previous findings obtained with DTG [11] and suggest that specific Sig-1R activation does not affect to the release of pro-inflammatory mediators after H/A.

### Characterization of Microglia/macrophages in the Ischemic Hemisphere – Effect of SA4503 Treatment

**Figure 5:**
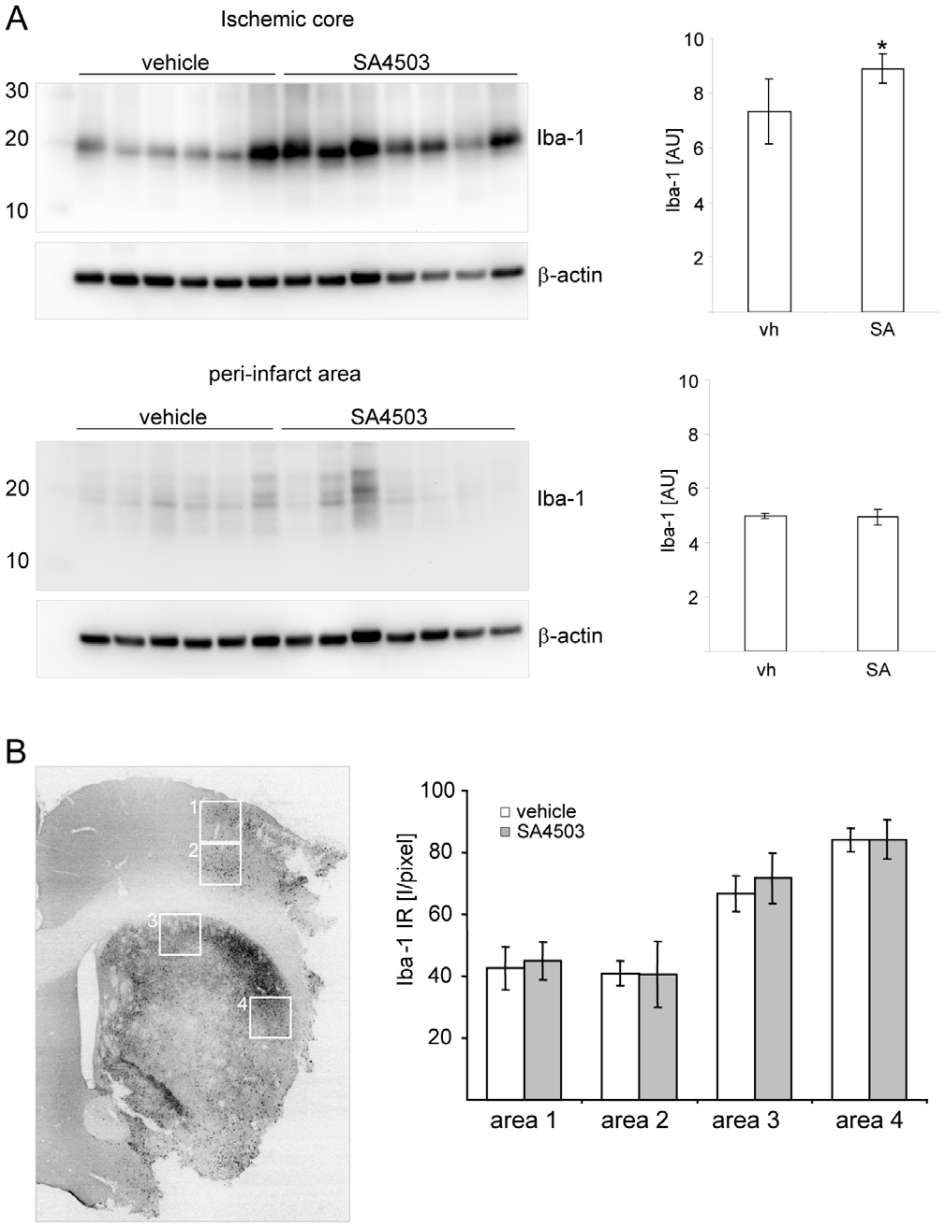
Iba1 levels in the infarct core after MCAO. (A) Western blot of samples from the infarct core and the peri-infarct area from SA4503 (0.5 mg/kg s.c.; n = 7) and vehicle treated rats (n = 6) and respective densitometric analyses (mean±SD, *p<0.05, t-test) at day 7 following MCAO. Note that each lane represents an individual animal. (B) Average Iba1 immunoreactivity in the ischemic hemisphere of vehicle and SA4503 rats on day 7 after tMCAo. Average Cy3 fluorescence intensity is presented per pixel (0 to 255) within the indicated areas considered as immunopositive Iba1, respectively (I/pixel; mean±s.d., n = 3 per treatment).

**Figure 6:**
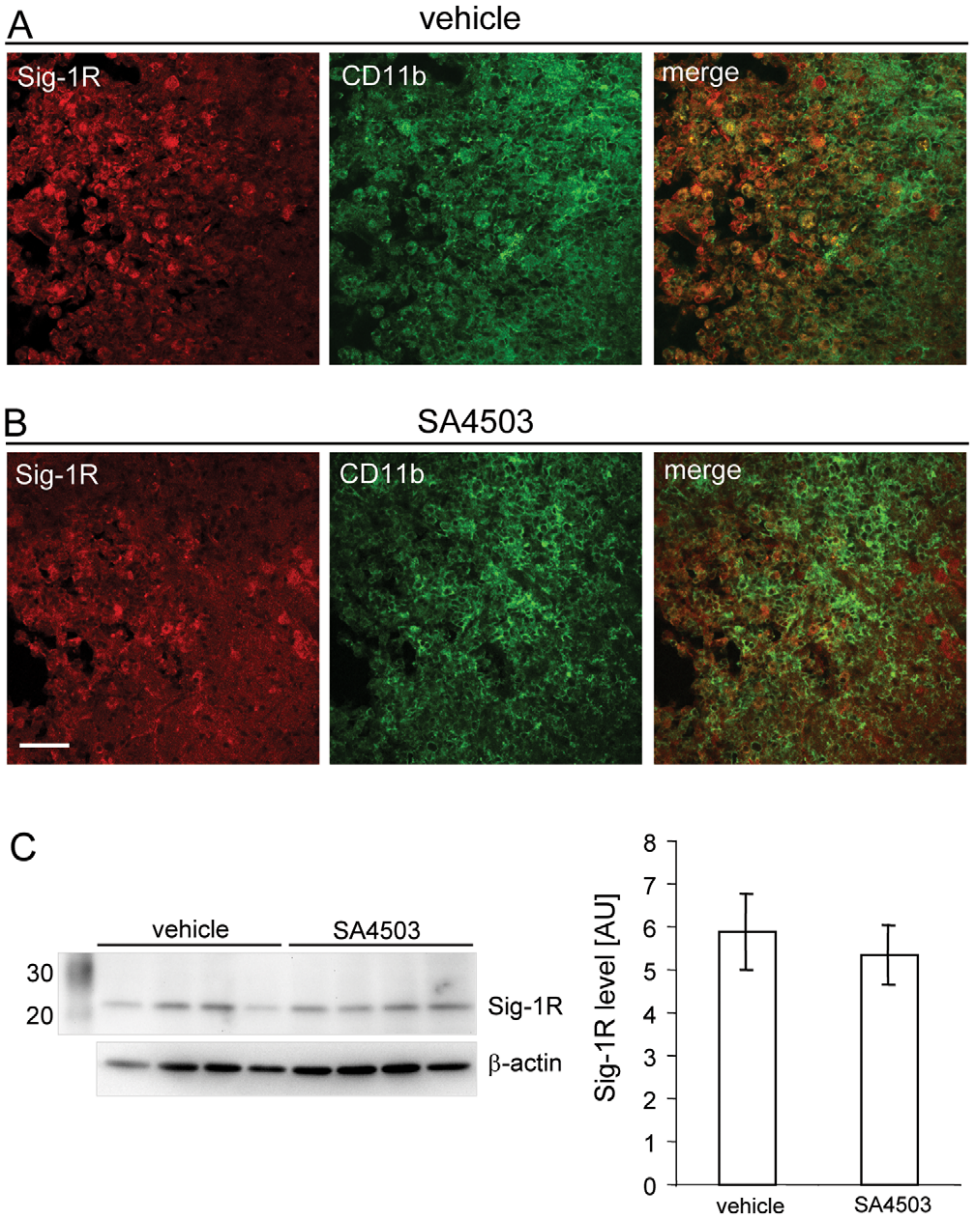
Expression of Sig-1R in OX-42^+^ cells in the ischemic hemisphere - Effect of SA4503 treatment. Sig-1R immunoreactivity in a vehicle (**A**) and SA4503 (**B**) treated animal after MCAO. In both animals Sig-1R (Cy3, red) is expressed in OX-42^+^ cells (Cy5, green) in the infarct core and adjacent peri-infarct area. Scale bars: 50 µm. (**C**) Western blot for the Sig-1R of samples from the ischemic infarct core from SA4503 (0.5 mg/kg s.c.) and vehicle treated rats and respective densitometric analyses (mean±SD, *p<0.05, t-test) at day 7 following MCAO. Each lane represents an individual animal and for densitometric analysis n = 8 rats were included for each treatment group.

The calcium binding protein Iba1 has been identified as a marker for activated microglia in the ischemic hemisphere [22]. We analyzed the levels of Iba1 in the infarct core from rats treated with saline or SA4505 after MCAO. As shown in Figure 5, we found significantly increased Iba1 levels in the infarct core of SA4503 treated animals (n = 8) but could not detect differences in the peri-infarct area between the treatment groups (Figure 5 A). In addition, the average Iba1 immunoreactivity, an indicator for the number of microglia/macrophages in the ischemic territory [20], did not differ significantly between the treatment groups indicating no difference in the number of activated microglia/macrophages in the ischemic territory (Figure 5 B). Taken together, our results demonstrate that SA4503 affects microglia/macrophage activation in the infarct core of rats subjected to tMCAO. We also evaluated if SA4503 treatment is associated with a change of the Sig-1R levels in respective animals. As shown in Figure 5 A, almost all OX-42^+^ cells in the infarct core were immunoreactive for Sig-1R. The Sig-1R immunoreactivity appeared in small cytoplasmic globules indicative of its location in lipid microdomains (Figure 6 B) [23]. No difference of Sig-1R levels was observed between the treatment groups determined by Western blot analysis (Figure 6 C).

**Figure 7:**
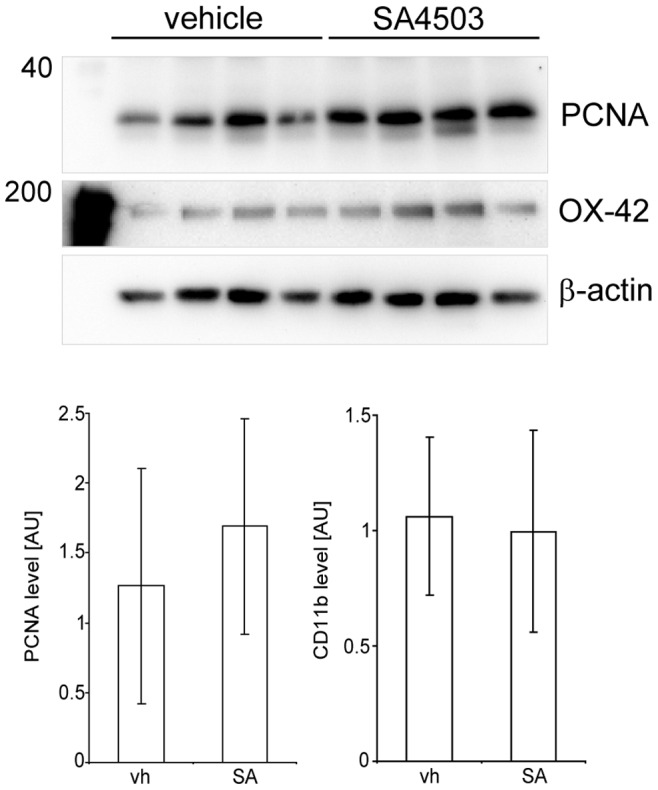
PCNA and OX-42 in the infarct core after MCAO. Representative Western blots of samples from the ischemic infarct core from SA4503 (0.5 mg/kg; n = 4) and vehicle treated (n = 4) rats at day 7 following MCAO. For densitometric analysis (mean±SD) n = 8 rats were included for each treatment group. Each lane represents an individual animal.

To evaluate if increased Iba1 levels are due to an increased proliferation of microglia/macrophages in the ischemic core of SA4503 treated animals we performed immunoblotting for Proliferating-Cell-Nuclear-Antigen (PCNA), a protein associated with cell proliferation. Figure 7 shows a representative Western blot for PCNA from four vehicle rats and four animals treated with SA4503 after MCAO. Analysis of the PCNA levels in the infarct core revealed no difference between the treatment groups (vehicle n = 8; SA4503 n = 8). Also, no difference in OX-42 levels was observed between the treatment groups in the infarct core (Figure 7). In summary, the results show that microglia/macrophage activation is independent of the changes in Sig-1R levels and that SA4503 treatment does not affect cell proliferation and the number of microglia/macrophages remains stable in the infarct core among the treatment groups.

## Discussion

Inflammation in the ischemic hemisphere significantly affects recovery of lost function following experimental stroke. The present study was conducted to assess if Sig-1R activation, by treatment with the specific agonist SA4503, affects the inflammatory response in the ischemic hemisphere. The findings we will discuss are: (1) Sig-1R activation does not affect the level of pro-inflammtory cytokines in the ischemic hemisphere and in microglia cultures after combined Hypoxia/Aglycemia. (2) SA4503 treatment enhances the levels of Iba1 in the infarct core after MCAO. (3) Effects of Sig-1R activation on neurodegeneration after MCAO.

### Effects of Sig-1R Activation on the Inflammatory Response After Stroke

Our study confirms an inflammatory response in the ischemic hemisphere which strongly depends on the activation of resident and invading cells and the expression of pro-inflammtory molecules in the ischemic territory [24]. Results corroborate that treatment with SA4503 for five consecutive days is not accompanied with changes of levels of pro-inflammtory cytokines in the infarct core and adjacent peri-infarct area. Data are in discrepancy to studies showing a suppression of pro-inflammatory cytokines upon Sig-1R activation [11], [13], [14], [25]. In particular, reduced levels of TNF-α were found in *in vitro* models using macrophages (RAW 264.7 cell line) treated with the Sig-1R agonist SR31747A [13] or isolated microglia treated with DTG [11] and acutely in the serum of mice treated with the Sig-1R specific agonist SSR125329A prior to a LPS challenge [14]. In additional *in vitro* experiments we compared the effect of SA4503 and DTG on nitrite production and the release of the pro-inflammatory cytokines TNF-α and IL-1β. While SA4503 did not affect inflammatory mediators, the application of DTG selectively reduced the levels of nitrite and TNF-α confirming data published previously [11]. Together, these findings make it plausible that treatment with SA4503 essentially does not affect levels of pro-inflammatory cytokines in experimental stroke models but rather is involved in the regulation of processes important for the beneficial effects observed at later time points after MCAO [18].

### Microglia/macrophage Response in the Ischemic Hemispshere

Microglia/macrophages but also other immune cells accumulate in the infarct core within the first days after experimental stroke [26]. Multiple functions of those cells include the formation of a preliminary scar to seal the necrotic infarct tissue. Consequently, the microglial scar may prevent the diffusion of detrimental molecules which otherwise may cause secondary damage to the unaffected tissue resulting in an expansion of the infarct and secondary neuronal death. In addition, microglia/macrophages may participate in the regulation of the entry of peripheral immune cells in the ischemic territory.

Previous studies have shown that highly proliferative Iba1/NG2 chondroitin sulfate proteoglycan positive microglia/macrophage with phagocytotic properties are present in the infarct core [27]. We found that treatment with SA4503 causes a significant increase of Iba1, a specific marker of microglia/macrophage activation, in the infarct core confirming previous studies showing elevated Iba1 levels in the ischemic core after MCAO [22]. Iba1 has been identified as a calcium binding protein belonging to the EF-hand protein family [28]. In addition, its colocalization with F-actin upon stimulation with M-CSF in MG5 microglial cells indicates its involvement in mechanisms of cell motility and phagocytosis [29]. Activation of the Sig-1R together with elevated levels of Iba1 in microglia/macrophages in the ischemic core of SA4503 treated animals might be important for the intracellular calcium homeostais [5], [30], [31]. However, it remains to be determined if the proposed mechanism contributes to motility of activated microglia/macrophages by inhibition of processes such as membrane ruffling and thus filopodia movements [32]. Also, Iba1 only serves as an indirect marker protein of microglia/macrophage activation and can not be used to differentiate microglia/macrophage populations which are involved in different processes such as the expression of trophic factors, phagocytosis or the involvement in the inflammatory response after stroke.

In addition, our results show that activation of the Sig-1R does not change the levels of PCNA, a general and established marker protein associated with cell proliferation [32], in the ischemic hemisphere indicating that the number of proliferating microglia/macrophages are unaffected by SA4503 treatment. Similar levels for OX-42 also indicate that the average number of cells is similar among the treatment groups and that SA4503 treatment does not affect the number of microglia/macrophages in the ischemic territory, although we can not exclude that the overall distributon of immune cell populations expressing OX-42 has been changed by the treatment. Our findings are different to results obtained by treatment with the unspecific Sig-1R agonist DTG decreased the number of OX-42^+^ cells in the ischemic territory [33]. The reduction in the number of OX-42^+^ cells, however, might be due to a reduced infarct size as well as different experimental designs.

### Neurodegeneration and Activation of Sig-1R

Our findings show that infarct volume is not affected by SA4503 treatment. Our results also indicate that treatment with SA4503 after experimental stroke requires a prolonged period of treatment time to achieve a significant improvement of lost neurological function [18]. Our findings are in contrast to previous investigations showing that delayed administration of the non-selective Sig-1R agonist 1,3-di-o-tolyguanidine (DTG) significantly decreases neurodegeneration after MCAO [12], [34]. Hence, the discrepancy might not be contradictory and be explained by different treatment windows and modes of actions of the compounds. 1,3-dio-tolylguanidine (DTG) is an unspecific sigma receptor agonist with similar binding affinity to the sigma-1 receptor and the sigma-2 receptor [35]. Therefore, further studies are needed to investigate if the short term neuroprotective effects after MCAO are mediated via activation of the sigma-1 receptor [36]. DTG was administered 24 h after MCAO, we started treatment at day two following MCAO with the rationale not to effectuate cell death mechanisms rather than mechanisms of recovery in the ischemic territory [18].

In addition, 1,3-di-o-tolyguanidine is known for an induction of hypothermia in rats which is partially inhibited by specific sigma receptor antagonists [37] and neuroprotective effects might be due to hypothermia induced in treated rats. Although we have not tested body temperature during the treatment period, it might have been that treatment with SA4503 has induced hypothermia. Nevertheless, beyond the period of neuroprotection hypothermia does not affect mechanisms involved in neuronal cell death. Therefore, it is likely that hypothermia induced by SA4503 treatment has no effect on recovery of lost neurological function after MCAO.

In conclusion, treatment with SA4503 had no effect on levels of pro-inflammatory cytokines in the infarct core and adjacent peri-infarct region but resulted in a significant increase in Iba1 expression in the infarct core. We conclude that treatment with SA4503 does not directly affect the classic inflammatory response in the ischemic hemisphere following experimental stroke but may be involved in mechanisms of microglia/macrophage motility and phagocytosis.
